# Relationship between’ patient’s rights charter’ and patients’ satisfaction in gynecological hospitals

**DOI:** 10.1186/s12913-016-1679-9

**Published:** 2016-09-07

**Authors:** Fereshteh Farzianpour, Abbas Rahimi Foroushani, Niusha Shahidi Sadeghi, Saeede Ansari Nosrati

**Affiliations:** 1 Health Management & Economic Department, School of Public Health, Tehran University of Medical Sciences, Tehran, Iran; 2 Epidemiology and Statistic Department, School of Public Health, Tehran University of Medical Sciences, Tehran, Iran; 3 Health care management, Health Management & Economic Department, School of Public Health, Tehran University of Medical Sciences, Tehran, Iran; 4 Department of Health Management and Economics, School of Public Health, Tehran University of Medical Sciences, Tehran, Iran

**Keywords:** Patient’s rights charter, Patient’s rights, Patient’s satisfaction, Gynecological hospitals

## Abstract

**Background:**

Patient’s satisfaction with hospital services is one of the most important indicators of efficiency and quality of services of different hospital wards.

**Methods:**

This cross-sectional descriptive analytical study was conducted in 2015. The study population included patients in gynecological hospitals of TUMS, and by using questionnaires; data were collected from 304 patients. Statistical analysis was performed using the SPSS 22.

**Results:**

The rights of patients were mainly observed through “quality of care” (Mean ± SD: 9.65 ± 2.62), “knowing the charges and the right to complain”; (Mean ± SD: 6.00 ± 2.5) “presence of an active system to handle complaints of patients in the hospital and explanation of the error that occurred during service provision to patients by the wrongdoer” is the lowest (7.5 ± 2.62). It was found that patients’ satisfaction is below the mean and its different aspects are higher than the mean level. However, the services of physicians and feeding recorded the highest and lowest satisfaction, respectively (19.4 ± 4.25, 20.77 ± 4.39). The mean score of satisfaction of patients admitted with nursing physical care was 24.5 ± 6.2.

**Conclusion:**

Overall, patients’ satisfaction with hospital services was close to the mean. Deficiencies and grievances should be resolved with a correct measure.

**Electronic supplementary material:**

The online version of this article (doi:10.1186/s12913-016-1679-9) contains supplementary material, which is available to authorized users.

## Background

Members of a society are committed to preserving and respecting the dignity of human beings [1]. People in every part of the health spectrum, have rights. When their rights are respected by others, especially health care providers, they will be more satisfied and secured [2, 3]. The rights of a patient includes the tasks that a medical center and the treatment team are obliged to implement and abide to for the physical, mental, spiritual and social legitimate needs embodied as standards, rules and regulations of therapy [4]. Dramatic changes have occurred in the way health services are received in the past few decades; for years, staff of organizations providing health services thought that they can make the best decision about patients, regardless of the their rights. But now the situation has changed and patients have expectations and demand for their rights [5]. Most health care providers are in a position that allows them determine patients’ fate, and in some cases, they behave like fathers. Although they see patients as intellectually mature and know they are allowed to decide on their own, but in the real sense, they do what they prefer [6]. Economic pressures, modern medicine and patients’ expectations are important factors that affect the identification of patients’ rights. Thus, multiple factors necessitated the development of principles dependent on support from individuals and health care and lay emphasis on patients’ rights which supports the present research [7]. An emphasis on basic human rights in health care, especially respect for patient’s dignity as a human becomes important when the patient’s vulnerability easily exposes him to violations and loopholes of the health and social system. The current era characteristics include not only the individual but social dimension of patients’ rights [8–10]. For this purpose, the healthcare system in most countries, has developed a charter for patient’s rights, to establish the rights of health care recipients and promote the ethical aspects of health care in one of the most important areas of health services, that is, treatment, and make it known to the executive levels to implement its provisions [11] which includes defending one’s rights in order to protect the sanctity and dignity at the time of illness to ensure strength of body and soul, and health care in the event of illness without age, sex discrimination and possessing sufficient financial power [12–20]. When patients are hospitalized, hospitals are obliged to submit this charter to the patients, to familiarize them with their rights [21–24].

Consumers are valuable sources of information to judge the quality of care services and their opinions feedback can be effective in organizing the hospital systematically. On the other hand, due to the importance of women in population growth, and the limited studies in this field, and also due to the need for a homogeneous population in terms of sex and disease, and the personnel taking care of patients who are women, the women were selected.

In this regard, the present study investigated the relationship between observance of patient’s rights charter and patient’s satisfaction with hospital inpatient services in specialized and ultra-specialized women’s hospitals of the Medical University of Tehran.

## Methods

This cross-sectional descriptive analytical study was conducted in the inpatient wards of all gynecological hospitals of the University of Medical Sciences. This research project approved in 2015 with code number 25831 and with the support of Research Deputy of Tehran University of Medical Sciences and Health Services. Data were collected by questionnaires given to patients of inpatient wards in gynecological hospitals of Tehran University of Medical Sciences in 2015. The research population consisted of the women’s comprehensive hospitals of MirzaKochakKhan and Arash. The number of sample was obtained from the correlation coefficient so that at 0.95 % confidence level and 0.90 % test power, if the correlation coefficient is 0.2 or more, it is statistically significant. The number of sample was obtained by the following formula:

$$ \mathrm{r} = 0.2;\mathrm{w} = 0.203 $$

$$ n=\frac{{\left({Z}_{1-\alpha /2} + {\mathrm{Z}}_{1\hbox{-} \mathrm{B}}\ \right)}^2}{W^2}+3 $$

$$ n=\frac{{\left(1.96+1.65\right)}^2}{(0.203)^2}+3=320 $$

For samples selection, first, the list of gynecological hospitals of Tehran University of Medical Sciences was provided with a number of hospitalizations per day. Patients were randomly selected from hospitalized patients in various wards. Data collection tools included two questionnaires whose validity and reliability were determined in previous studies [25]. The first questionnaire was developed in two parts, the first part included demographic variables while the second part included 13 paragraphs as follows: Information on patient’s rights (Likert from 1 = very unsatisfied to 5 = very satisfied), providing personal information including the full name of members of the care team and presenting professional information which includes the occupation, responsibility of the care team members to the patient, or his alternative decision-maker when admitted at the medical center, patient’s right to receive respectful and non-discriminatory diagnostic-medical services, delivering good care, providing sufficient information about the disease, treatment methods and common complications, patient’s and the alternative decision-maker’s access to health care team during hospitalization and after discharge from the hospital in an understandable language, accountability of the care team for the questions of patient or alternative decision-maker about disease and treatment, the patient’s right to choose and decide, authority and independence, the right to replace doctor and refuse treatment and its consequences, patient’s access to his/her medical records and information on the content, maintaining patient’s privacy, information confidentiality and secrecy, patient’s training, patient’s right for consideration of the complaints and expression of medical error, presence of an active system to handle complaints by patients in the hospital, explanation of the error that occurred during service provision to patients by the wrongdoer, and knowing about the charges and the right to complain, in the form of 90 multiple-choice questions in Likert scale. It assesses the level of respect for patients’ rights charter. The second questionnaire includes the following 6 parts which assessed patient’s satisfaction, and its validity and reliability have been confirmed in previous studies [25, 26]: satisfaction of admitted patients with nursing physical care Likert from 1 = very unsatisfied to 5 = very satisfied), admitted patients’ satisfaction with nursing psychological and mental care, patients’ satisfaction with medical services, patients’ satisfaction with feeding, patients’ satisfaction with the physical environment, as well as facilities and reception services which are determined based on past studies and literature review. Finally, the questionnaires (Additional files 1 and 2). were distributed among the patients in the hospitals studied, according to the mentioned method. Analysis of data was performed using SPSS 22 statistical software and descriptive statistics together with Pearson’s correlation coefficients and, two-sample *T*-test, as well as ANOVA tests.

## Results

In the current study, an age group of 27–35 years (50.7 %), married (93.8 %), those with bachelor’s degree or higher (54.27 %) and duration of stay at the time of completing the questionnaire (40.4 %) had the largest volume of the sample.

Most of these patients (68.8 %) had a history of one hospitalization. 43.7 % of them had a history of hospitalization in other hospitals and 64.4 % in public hospitals (Table 1).

**Table 1:** Frequency distribution of the people who completed the questionnaire according to demographic the questionnaire according to demographic variables

Variable	Number	Percentage
Age group		
17-26	74	24.3
27-35	154	50.7
36-57	76	35
Marital status		
Single	19	6.25
Married	285	93.8
Level of education		
Elementary	35	11.51
diploma	104	34.21
Bachelor and higher	165	54.27
Job type		
Employed(teacher + others)	57	18.8
Unemployed(housewife)	247	81.2
Reason for hospitalization		
giving birth(delivery)	42	13.8
Medical	224	73.3
Surgery	38	12.5
Number of hospitalization days		
1-2	115	38.1
3-5	122	40.4
6-60	65	21.5
Number of hospitalizations		
0(outpatient)	27	8.9
1(inpatient)	209	68.8
2-7(inpatient)	68	22.4
Type of insurance		
Public social security	197	64.8
Public health servise	83	27.3
Private	24	7.9
Supplemental Insurance		
Yes	229	75.3
No	75	24.7
Referral to other hospitals		
Yes	131	43.7
No	163	56.3
Number of referral to other hospitals		
Less than 2 times	73	54.1
Between 2-4	55	40.7
More than 4 times	7	5.2
Economic status		
High	83	27.3
Medium	197	64.8
Low	24	7.9

The patients’ view on “the right to replace the doctor and refuse treatment and its consequences” on the patient’s rights charter with mean score of 10.12 ± 3.94 is more than low and less than neither low, nor high.

The patients’ view on “access to his/her medical records and information on the content” of the patient’s rights charter with a mean score of 2.41 ± 1.27 is more than neither low, nor high and less than high. View of the patients on “maintaining patient”s privacy“ of the patient’s rights charter with mean score of 17.84 ± 4.99 is more than neither low, nor high and less than high. The patients’ view on “information confidentiality and secrecy” of the patient’s rights charter has a mean score of 4.67 ± 1.48, is more than neither low, nor high and less than high (Table 2 and Additional files 1 and 2).

**Table 2:** Average of the various aspects of patients' rights in the hospital under study

Dimension	Number	Least	Most	Total	Mean	SD
Notification	304	6	28	3382	11.13	5.05
Patient’s right to receive diagnostic-medical services	304	5	18	3037	9.99	2.56
Quality of care	304	3	15	2935	9.65	2.62
Providing adequate information on the disease	304	9	44	6330	20.82	7.28
Medical team accountability for the questions of patient	304	2	10	1662	5.47	2.03
Patient’s right to decide	304	7	26	3749	12.33	4.72
The right to replace the doctor and refuse treatment	304	6	23	3076	1.12	3.94
Patient access to records	304	1	5	734	2.41	1.27
Maintaining patient’s privacy	304	9	32	5423	17.84	4.99
Confidentiality of information	304	2	10	1421	4.67	1.48
Patients’ straining	304	4	20	3080	10.13	3.61
Knowing about the charges and the right to complain	304	4	19	1824	6.00	2.5
Patient’s right to consider the complaints and expression of medical error	304	5	15	2280	7.5	2.62

Patients’ satisfaction with “nursing physical care” with mean score of 24.5 ± 6.2 is more than satisfied and less than neither satisfied nor dissatisfied. Patients’ satisfaction with “medical services” with mean score of 19.14 ± 4.25 is more than satisfied and less than neither satisfied nor dissatisfied (Table 3 and Additional files 1 and 2).

**Table 3:** Average of various aspects of satisfaction among hospitalized patients under study

Dimension	Number	Least	Most	Total	Mean	SD
Satisfaction of patients admitted with nursing physical care	304	10	41	7450	24.5	6.2
Satisfaction of patients admitted with nursing psychological care	304	11	46	8195	26.96	6.47
Satisfaction with medical services	304	8	32	5819	19.14	4.25
Satisfaction with feeding	304	7	32	6315	20.77	4.39
Satisfaction with amenities	304	11	52	8992	29.58	7.38
Satisfaction with admission	304	6	30	4905	16.13	4.02

The Kolmogorov-Smirnov test was used to test normality of the satisfaction level. Since test statistic = 1.37 and *P >* 0.05, the distribution of this score is not normal, hence the correlation coefficient of Spearman, Mann–Whitney or Kruskal-Wallis were used to examine the effect of demographic factors on the median of the score.

Satisfaction is different in groups with different causes of hospitalization (*P-*value < 0.001). ‘Delivery’ and ‘surgical’ groups have the highest dissatisfaction and satisfaction, respectively.

Satisfaction is different in groups with different insurances (*P-*value < 0.001) (Table 4 and Additional files 1 and 2). ‘Other insurances’ and ‘medical services’ groups recorded the highest dissatisfaction and satisfaction, respectively.

**Table 4:** Analysis of the statistic test for the relationship between demographic characteristics and patients’ level of satisfaction with health care services

	Group	Number	Average rank	Test result
Satisfaction	Employed(teacher + other)	57	159.84	Z = −0.7
	Unemployed(housewife)	274	150.81	Z = 0.48
Satisfaction	Single	19	148.63	Z = −0.2
	married	285	152.76	Z = 0.843
Satisfaction	With supplemental insurance	75	149.52	Z = −0.34
	Without supplemental insurance	229	153.48	P = 0.74
Satisfaction	Age group of 17-26	74	146.7	Chi-square = 7.21
	Age group of 27-35	154	164.94	df = 2
	Age group of 36-57	76	132.89	P = 0.027
Satisfaction	Elementary	35	35	Chi-square = 4.68
	diploma	104	104	df = 2
	Bachelor an higher	165	165	P = 0.096
Satisfaction	Delivery hospitalization	42	190.38	Chi-square = 19.6
	Medical hospitalization	224	153.7	df = 2
	Surgical hospitalization	38	103.63	p < 0.001
Satisfaction	Hospital stay: 1–2 days	115	138.25	Chi-square 4.85
	Hospital stay: 3–5 days	122	156.13	df = 2
	Hospital stay: 6–60 days	65	166.25	P = 0.088
Satisfaction	number of hospitalization: 0*	27	155.59	Chi-square = 0.48
	Number of hospitalization: 1**	209	154.2	df = 2
	Number of hospitalization: 2-7**	68	146	P = 0.79
Satisfaction	Public social security insurance	197	161.46	Chi-square = 26.63
	Public medical services insurance	83	115.16	df = 2
	Other insurances(Private)	24	208.08	p < 0.001
Satisfaction	Economic status appropriate	83	115.16	Chi-square = 26.63
	Relatively good	197	161.46	df = 2
	Inappropriate	24	208.08	p < 0.001

*Outpatient, **Inpatient

Spearman correlation test was used to assess the relationship between respect for patient’s rights charter and patient’s satisfaction with hospital services, and the results are as follows. Notification about patient’s rights, Patient’s right to receive diagnostic-medical services, Quality of care, providing adequate information on the disease, Medical team accountability for the questions of patient, Patient’s right to decide, choose, authority and independence, Patient training, Knowing about the charges and the right to complain, Presence of an active system for complaints of patients(*P-*value < 0.01) and The right to replace the doctor and refuse treatment (*P-*value < 0.05), Patient’s access to medical records and contents (*P-*value < 0.001), Maintaining patient’s privacy, Confidentiality of information (*P-*value < 0.251) (Table 5 and Additional files 1 and 2).

**Table 5:** Statistical test results of the relationship between respect for patient’s rights charter and patient’s satisfaction with health care services

hospital services patient’s satisfaction with	Dimensions of patient’s rights charter	Test results
	Notification about patient’ sright	Correlation coefficient = −0.249
		P-value <0.01
	Patient’s right to receive diagnostic-medical services	Correlation coefficient = −0.304
		P-value < 0.01
	Quality of care	Correlation coefficient = −0.532
		P-value < 0.01
	Providing adequate information on the disease	Correlation coefficient = −0.284
		P-value < 0.01
	Medical team accountability for the questions of patients	Correlation coefficient = −0.354
		P-value < 0.01
	Patient’s right to decide, choose, authority and independence	Correlation coefficient = −0.206
		P-value < 0.01
	The right to replace the doctor and refuse treatment	Correlation coefficient = −0.147
		P-value < 0.05
	patient’s access to medical records and contents	Correlation coefficient = −0.206
		P-value < 0.001
	Maintaining patient’s privacy	Correlation coefficient = −0.477
		P-value < 0.01
	Confidentiality of information	Correlation coefficient = −0.066
		P-value <0.251
	Patient training	Correlation coefficient = −0.370
		p-value < 0.01
	Knowing about the charges and the right to complain	Correlation coefficient = −0.150
		P-value < 0.01
	Presence of an active system for complaints of patients	Correlation coefficient = −0.260
		P-value < 0.01

## Discussion

According to results of the age group of 27–35 years (50.7 %), those married (93.8 %), those with Bachelor’s degree or higher (54.27 %) and duration of stay at the time of completing the questionnaire (40.4 %) had the largest volume of respondents.

Most of these patients (68.8 %) had a history of one hospitalization. A total of 43.7 % of them had a history of hospitalization in other hospitals and 64.8 % in public hospitals. This will help in interpreting the research findings. Most of them had social security insurance (64.8 %) and none had supplemental insurance (75.3 %). In this study, the highest amount of compliance with patient’s rights charter was related to the “quality of care”. Also, patients’ view on observance of “knowing the charges and the right to complain” and “presence of an active system to handle complaints of patients in the hospital, and explanation of the error that occurred during service provision to patients by the wrongdoer” had the lowest value among 13 dimensions of the patient’s rights charter.

Patients’ view on observance of “providing sufficient information about the disease, treatment methods and common complications, patient’s and the alternative decision-maker’s access to health care team during hospitalization and after discharge from the hospital in an understandable language”, with mean score of 6.00 ± 2.5 are more than neither low nor high and less than high (Additional files 1 and 2). In their study, Nasiriyani et al. [23] noted that patients considered improper accountability of doctors and nurses, as the reason for not asking questions. Also, Bazmi et al. [24] reported the lowest score for the right to receive necessary information on possible side effects, as well as other treatment options and participation in the final selection of treatment. This is due to the instruments used or the year of the study, because during the study of Bazmi et al. [24], patient’s rights charter had 5 dimensions, but now it has 13 dimensions.

Patients’ view on observance of “information confidentiality” of the patient’s rights charter with mean score of 2.23 is more than neither low nor high and less than high. According to Bazmi et al. [24], patients assigned the highest score to confidentiality of information with the doctors and the medical team. This can be due to the instruments used or the year of the study, because in Bazmi’s study, patient’s rights charter had 5 dimensions, but presently, there are13 dimensions.

Patients’ view on observance of “informing about patient’s rights, providing personal information including full name of the care team members and presenting professional information including the occupation, responsibility of the care team members for the patient, or his alternative decision-maker when admitted at the medical center”, with mean score of 1.85 is more than low and less than neither low nor high. Nasiriyani et al. [23] showed that the least observance of the rights of study participants is when care team members are not introduced to patients, which is partly consistent with this study. Anbari et al. [25] showed that the highest mean score for patients’ rights at “the time of admission” is for “mostly observed”.

Hospitalized patients’ satisfaction with “nursing physical care” and “nursing psychological care” is more than average. Most psychological satisfaction with nursing care in the present study was to deal respectfully with patients which is consistent with the study of Ghanbari and colleagues [22].

Hasaniyan [21] showed that most of the patients’ dissatisfaction with nurses is in non-allocation of sufficient time, in order to give information on the plan and procedures in progress. They argued that patients’ anxiety can be reduced by speaking to nurses and getting information which will make them feel more relaxed.

However, in the study of Hasaniyan [21], patients admitted to Ajay hospitals in the city of Tehran were more satisfied with the performance of doctors and nurses, and their dissatisfaction with the performance of nurses was twice more than that with the performance of doctors. Of course, in the study conducted by Nemati et al. [26], satisfaction with nursing care (72.3) was significantly more than that with medical services (57.6). These different results can be caused by the research environment and the tools used.

In this study, patients’ satisfaction with “Medical Services” is more than the average, and has the highest score with a little difference. In the study conducted by Nemati et al. [26], satisfaction with “Medical Services” was reported to be more than the average which is consistent with the current study.

The least satisfaction with medical services is the availability of doctor when needed and also, with a little difference in medical explanation of disease. Also, in the study of Nemati et al. [26], the least satisfaction was with access to medical staff when needed.

Ghanbari et al. [22] also showed that the lowest satisfaction with medical services (12 %) was medical explanation in the field of disease.

The lowest score of patients’ satisfaction in this study, with minor differences from the other axes, is in the “feeding” axis.

In general, the analysis of results of the current study shows that patients’ satisfaction in different axes is in one level and higher than average. However, physicians’ services and feeding axes had the highest and lowest satisfaction, respectively.

In this study, the mean score of satisfaction was below average. Nemati [26] showed that the percentage of satisfaction with total service was at a quite satisfied level which is inconsistent with the current study. Perhaps, this difference is as a result of the different dimensions measured in the two studies; 7 and 3 dimensions were studied in the present study and Nemati’s study, respectively.

Mean score of patient’s rights charter observance (2.09 %) was almost low. The results of this study confirmed that of Aghighi et al. [27] who reported that the patient’s rights charter observance was equal to 8.75 %. Asterki et al. [28] also showed that in general, patients know the observance of rights charter on average. Anbari et al. [25] also stated that patient’s rights observance in surgical wards is not appropriate in patients’ view Vakili et al. [29] reported that the patient’s rights charter observance was equal to 2.63 % which is inconsistent with the current study. This difference can be due to the instruments used or the year of the study, because in Vakili’s study, patient’s rights charter had 5 dimensions, but in the present study, it is 13 dimensions.

Patients’ rights observance is the most important part of ethical issues that requires consideration. If the rights of patients and their families are respected by therapists, it has the advantage that patients can be aware of the course of treatment and make sure that hospitalization is effective [25].

Satisfaction is different in different age groups (*P-*value = 0/027). Age group of 27.35 years had the highest dissatisfaction with inpatient services. According to a study conducted at Aja hospitals, old people had higher satisfaction mean scores.

Kavari, Garosi, Mack and Nemati [26, 30–32] conducted separate studies and concluded on the same results, which means that younger patients are less satisfied than middle-aged and older people because of higher awareness of this young and active age group, of their rights.

Satisfaction is different in groups with different causes of hospitalization (*P-*value < 0.001). “Delivery” and “surgical” groups had the highest dissatisfaction and satisfaction, respectively.

Satisfaction is different in groups with different insurances (*P-*value < 0.001). “Other insurances” and “medical services” groups had the highest dissatisfaction and satisfaction, respectively. Nemati et al. [26] showed that satisfaction of those with insurance is higher than those without insurance.

There was no statistical relationship between satisfaction and other demographic variables. Results studies by Hasaniyan, Yaghmaei and Akbari [21, 33, 34] are entirely consistent with the results of the present study. But the study of Mack [32] showed that patients with a history of hospitalization were more satisfied with the service received.

At *P <*0.001 level, patient’s rights charter observance in patients’ perspective and dissatisfaction scores were negatively correlated. This means that by increasing the observance of the patient’s rights charter, satisfaction score increases. In the study of Vakili et al. [29], patients’ satisfaction with the hospital was significantly correlated with the level of patients’ observance in all domains, which is consistent with the present study.

To find more accurate results for the relationship between patient’s rights charter and patient’s satisfaction with health care services we suggest to conduct other studies such as intervention or clinical studies in which the effect of intervention on patient’s right character on patient’s satisfaction can be estimated after controlling for confounding factors.

## Conclusion

According to the observance of patients’ rights and its relationship with patients’ satisfaction with hospital, to increase patients’ satisfaction and improve service delivery to them, patient’s rights compliance is necessary. And one of the main strategies to increase patients’ rights compliance is to increase their awareness of patients’ rights charter.

With regards to correlation between patients’ satisfaction and their rights charter axes, the highest correlation belonged to the “quality of care” and with a little difference from “maintaining patient’s privacy”; also, the lowest correlation belonged to the “right to replace the doctor and refuse treatment and its consequences”. In addition, there is no significant correlation between patients’ satisfaction and “information confidentiality and secrecy ”of the patient’s rights charter.

## Additional files

Additional file 1: Questionnaire for Patient’s rights charter. (DOCX 18 kb) Additional file 2: Questionnaire for patient,s satisfaction. (DOCX 46 kb)

## Abbreviations

ANOVA: Analysis of variance
SD: Standard deviation
TUMS: Tehran University of medical sciences
