# Supplementary material for: Relationship between’ patient’s rights charter’ and patients’ satisfaction in gynecological hospitals
Source: BMC Health Serv Res. 2016 Sep 7;16(1):476. doi: 10.1186/s12913-016-1679-9 (PMC5015240; doi:10.1186/s12913-016-1679-9)
Supplement: Additional file 1: — Questionnaire for Patient’s rights charter. (DOCX 18 kb) [file 12913_2016_1679_MOESM1_ESM.docx]

(Supplementary file 1)

**Appendix 1**

Iranian patients’ bill of rights, the Ministry of Health and Medical

Education, 2015 ,Tehran-Iran

1) The patient has the right to considerate and respectful care regardless of race,

culture and religion.

2) The patient has the right to know the identity of physicians, nurses and others

involved in his/her care.

3) The patient has the right to obtain from physicians understandable information

concerning his/her diagnosis, treatment and prognosis, except in emergencies

that would delay the provision of urgent treatment that may threaten his/her life.

4) The patient has the right to request information related to specific diagnostic

procedures and treatments, the risks and possible alternatives.

5) The patient has the right to refuse treatment and/or be referred to another

hospital except in cases of threats to community health.

6) The patient has the right to expect that all communications and records pertaining

to his/her care will be treated as confidential by the hospital except in cases of

public health hazards, when reporting is permitted or required by law.

7) The patient has the right to every consideration to privacy and confidentiality.

8) The patient has the right to accessibility to physicians and other health care

providers during hospitalization, transition to other institutions and discharge.

9) The patient has the right to consent or decline to participate in research studies

without any threatening consequences.

10) The patient has the right to be informed of hospital policies, charges and insurance

coverage in addition to care providers’ expertise in the current hospital and the

hospital to be referred to.

Perspective on patients’ rights 15

Nursing
