# Supplementary material for: Relationship between’ patient’s rights charter’ and patients’ satisfaction in gynecological hospitals
Source: BMC Health Serv Res. 2016 Sep 7;16(1):476. doi: 10.1186/s12913-016-1679-9 (PMC5015240; doi:10.1186/s12913-016-1679-9)
Supplement: Additional file 2: — Questionnaire for patient,s satisfaction. (DOCX 46 kb) [file 12913_2016_1679_MOESM2_ESM.docx]

**Questioner 1**

Part I: Personal Information
1. Date of Birth: ................
2. Sex: Female € Male €
3. Level of Education: illiterate € primary€ and middle schools€ and Diploma€ university€

4. Employment situation: unemployed workers€ retire€: housewife€ university € student€ soldiers €staff€………………………………
5. Marital status: Single€ Married€
6. The reason for hospitalization in this section (diagnosis)?
7. The length of stay in hospital .....................
8. Read admitted to the hospital ......................
9. Type of insurance coverage? ................
10. Do you supplement insurance? No € Yes€
11. If the answer is yes supplemental insurance? .....................
12. History of hospitalization of others? No € Yes€
13. If the answer is yes, please answer the questions below?
13-1 less than time€ 2 € 4-2 € more than 4 times €
13-2- in what hospital? Public€ private€ Social Security € other€ army €

**2. Information about patient rights**

| 1. On arrival, you are the nurses, have introduced themselves by name? | | | | | | | | | | | | | | | | |
| --- | --- | --- | --- | --- | --- | --- | --- | --- | --- | --- | --- | --- | --- | --- | --- | --- |
| Ever(1) | | | little(2) | | | | | | Not least so much(3) | | much(4) | Very much(5) | | |  | |
| 2. On arrival, you are part of the information understandable and simple language about the group provider such as a physician or physicians, nurses, and service, you have received? | | | | | | | | | | | | | | | | |
| Ever(1) | | | little(2) | | | | | | Not least so much(3) | | much(4) | Very much(5) | | |  | |
| 3. On arrival, you are part of language comprehensible and simple information on how to access services and health care and non-health sectors have received? | | | | | | | | | | | | | | | | |
| Ever(1) | | | | | little(2) | | | | Not least so much(3) | | much(4) | Very much(5) | | |  | |
| 4. On arrival you are part of the information understandable and simple language about the rules and hospital sectors such as hours of sleep, waking, eating and ... the treatment you have received? | | | | | | | | | | | | | | | | |
| Ever(1) | | | | | little(2) | | | | Not least so much(3) | | much(4) | Very much(5) | | |  | |
| 5. On arrival, you are the health care team about your professional profile includes job and task information you have provided? | | | | | | | | | | | | | | | | |
| 6. On arrival you are part of the information understandable and simple language about the “patient rights” of the section has received? | | | | | | | | | | | | | | | | |
| Ever(1) | | | | | little(2) | | | | Not least so much(3) | | much(4) | Very much(5) | | |  | |
| 7. On arrival, you are part of the healthcare team to inform you that this is a teaching hospital? | | | | | | | | | | | | | | | | |
| Ever(1) | | | | | little(2) | | | | Not least so much(3) | | much(4) | Very much(5) | | |  | |
| **Section3** | | | | | | | | | | | | | | | | |
| Patient's right to receive diagnostic services - respectful treatment and non-discrimination (in terms of gender, ethnicity, type of insurance, income, etc.) | | | | | | | | | | | | | | | | |
| 1. The treatment provided during their stay in the hospital you have been? | | | | | | | | | | | | | | | | |
| Ever(1) | | | | | little(2) | | | | Not least so much(3) | | much(4) | Very much(5) | | |  | |
| 2. The length of stay in the hospital in the past have come to feel that your dignity is insulted? | | | | | | | | | | | | | | | | |
| Ever(1) | | | | | little(2) | | | | Not least so much(3) | | much(4) | Very much(5) | | |  | |
| 3. You in the hospital diagnostic or therapeutic services without discrimination (gender, ethnicity, religion, type of insurance, inland ...) is presented? | | | | | | | | | | | | | | | | |
| Ever(1) | | | | | little(2) | | | | Not least so much(3) | | much(4) | Very much(5) | | |  | |
| 4-ever report that you feel that a certain type of health care due to their ethnicity or religion have been deprived of this facility? | | | | | | | | | | | | | | | | |
| Ever(1) | | | | | little(2) | | | | Not least so much(3) | | much(4) | Very much(5) | | |  | |
| 5. Thus far, by the hospital staff feel that religious beliefs or other beliefs offend you are? | | | | | | | | | | | | | | |  | |
| Ever(1) | | | | | little(2) | | | | Not least so much(3) | | much(4) | Very much(5) | | |  | |
| 6. In your opinion are respected in the hospital to the patient? | | | | | | | | | | | | | | |  | |
| Ever(1) | | | | | little(2) | | | | Not least so much(3) | | much(4) | Very much(5) | | |  | |
| 7. In the hospital medical staff treating the patient with an entourage respectful? | | | | | | | | | | | | | | |  | |
| Ever(1) | | | | | little(2) | | | | Not least so much(3) | | much(4) | Very much(5) | | |  | |
| 7. In the hospital medical staff treating the patient with an entourage respectful? | | | | | | | | | | | | | | |  | |
| Ever(1) | | | | | little(2) | | | | Not least so much(3) | | much(4) | Very much(5) | | |  | |
| **Section 4** | | | | | | | | | | | | | | |  | |
| The quality of care (providing diagnostic services - therapy soon as possible to the patient) | | | | | | | | | | | | | | |  | |
| 1. Do you think medical staff to efficiently carry out their duties? | | | | | | | | | | | | | | |  | |
| Ever(1) | | | | | | little(2) | | | Not least so | | much(4) | Very much(5) | | |  | |
| 2. During this hospital stay, easy access to staff when necessary in the fastest possible time have you had? | | | | | | | | | | | | | | |  | |
| Ever(1) | | | | little(2) | | | | | Not least so much(3) | | much(4) | | Very much(5) | |  | |
|  | | | | | | | | | | |  | | | | | |
| 3. In this hospital to diagnostic services - treatment in the quickest possible time was given to you? | | | | | | | | | | | | | | |  | |
| Ever(1) | | | | | little(2) | | | | Not least so much(3) | | much(4) | Very much(5) | | |  | |
| Provide sufficient information about diseases, therapies and pain, patient access to health care during hospitalization and after discharge from the hospital in a language comprehensible to the patient or substitute decision maker, he | | | | | | | | | | | | | | |  | |
| 1. The staff treat you in a language comprehensible and simple rules on access to services and health care and health outcomes have informed? | | | | | | | | | | | | | | |  | |
| Ever(1) | | | | | little(2) | | | | Not least so much(3) | | much(4) | Very much(5) | | |  | |
| Clinicians, you understand and simple language that during hospitalization how your healthcare team keep in contact with? | | | | | | | | | | | | | | |  | |
| Ever(1) | | | | | little(2) | | | | Not least so much(3) | | much(4) | Very much(5) | | |  | |
| 3. Your doctor can understand language and simple information about diseases, therapies, diagnostics and approval, test and diagnostics   That clinical understandable and simple language that must be done, has given you? | | | | | | | | | | | | | | |  | |
| Ever(1) | | | | | little(2) | | | | Not least so much(3) | | much(4) | Very much(5) | | |  | |
| 4. The treatment information in understandable and simple enough about diseases, therapies, diagnostics and approval, and recognize that clinical tests that must be conducted in a language comprehensible and easy to you have given? | | | | | | | | | | | | | | |  | |
| Ever(1) | | | | | little(2) | | | | Not least so much(3) | | much(4) | Very much(5) | | |  | |
| 5. Your health care provider information understandable and simple language about diseases, therapies, diagnostics and approval, and recognize that clinical tests that must be performed in a simple and understandable language, along with you? | | | | | | | | | | | | | | |  | |
| Ever(1) | | | | | little(2) | | | | Not least so much(3) | | much(4) | Very much(5) | | |  | |
| 6. The medical staff have sufficient information understandable and simple language about diseases, therapies, diagnostics and approval, test and Clinical diagnoses that should be understandable language and easy to take along are you? | | | | | | | | | | | | | | | | |
| Ever(1) | | | | | little(2) | | | | Not least so much(3) | | much(4) | Very much(5) | | |  | |
| 7. If your doctor to perform a certain type of tests and x-rays, such as MRI, CT scan has prescribed for you the staff, the information understandable and simple language requirements you have? | | | | | | | | | | | | | | | | |
| Ever(1) | | | | | little(2) | | | | Not least so much(3) | | much(4) | Very much(5) | | |  | |
| 8. Nurses about care after discharge, diet and activity levels associated with the diagnosis you have information in a language comprehensible and simple? | | | | | | | | | | | | | | | | |
| Ever(1) | | | | | little(2) | | | | Not least so much(3) | | much(4) | Very much(5) | | |  | |
| 9. If your doctor to perform a certain type of tests and x-rays, such as MRI, CT scan has prescribed for you the staff, the information needed to understand and simple language to accompany you have? | | | | | | | | | | | | | | | | |
| Ever(1) | | | | | little(2) | | | | Not least so much(3) | | much(4) | Very much(5) | | |  | |
| 10- Nurses about care after discharge, diet and activity levels associated with the diagnosis understandable and simple language to accompany you to have the information? | | | | | | | | | | | | | | | | |
| Ever(1) | | | | | little(2) | | | | Not least so much(3) | | much(4) | Very much(5) | | |  | |
| 11 - Doctor or physicians about the kind of care after discharge, diet and activity levels associated with the diagnosis and simple language understandable information you have? | | | | | | | | | | | | | | | | |
| Ever(1) | | | | | little(2) | | | | Not least so much(3) | | much(4) | Very much(5) | | |  | |
| Doctor or physicians about the kind of care after discharge, diet and activity levels associated with the diagnosis understandable and simple language to accompany you to have the information? | | | | | | | | | | | | | | | | |
| Ever(1) | | | | | little(2) | | | | Not least so much(3) | | much(4) | Very much(5) | | |  | |
| 3. Doctor or doctors about side effects common, understandable and simple language the information you have? | | | | | | | | | | | | | | | | |
| Ever(1) | | | | | little(2) | | | | Not least so much(3) | | much(4) | Very much(5) | | |  | |
| Nurses incapacitating disease, you have the information in an understandable and simple? | | | | | | | | | | | | | | | | |
| Ever(1) | | | | | little(2) | | | | Not least so much(3) | | much(4) | Very much(5) | | |  | |
| 15. The physician or physicians about common side effects, along with the information you have given language understandable and simple? | | | | | | | | | | | | | | | | |
| Ever(1) | | | | | little(2) | | | | Not least so much(3) | | much(4) | Very much(5) | | |  | |
| 16. Is nurses about common side effects, along with the information you have given language understandable and simple? | | | | | | | | | | | | | | | | |
| Ever(1) | | little(2) | | | | | Not least so much(3) | | | | much(4) | Very much(5) | | |  | |
| 17. Doctor or physicians about rare disease, you have the information in an understandable and simple? | | | | | | | | | | | | | | | | |
| Ever(1) | | little(2) | | | | | Not least so much(3) | | | | much(4) | Very much(5) | | |  | |
| 18.Doctor or physicians about rare diseases, along with the information you have given language understandable and simple? | | | | | | | | | | | | | | | | |
| Ever(1) | | little(2) | | | | | Not least so much(3) | | | | much(4) | Very much(5) | | |  | |
| 19. Nurses rare complication of the disease, you have the information in an understandable and simple? | | | | | | | | | | | | | | | | |
| Ever(1) | | little(2) | | | | | Not least so much(3) | | | | much(4) | Very much(5) | | |  | |
| 20. Nurses rare complication of the disease, along with the information you have given language understandable and simple? | | | | | | | | | | | | | | | | |
| Ever(1) | | little(2) | | | | | Not least so much(3) | | | | much(4) | Very much(5) | | |  | |
| 21. The staff treat you in a language comprehensible and simple that after discharge from the hospital how to keep in contact with your health care team? | | | | | | | | | | | | | | | | |
| Ever(1) | | little(2) | | | | | Not least so much(3) | | | | much(4) | Very much(5) | | | | |
| **Section 5** | | | | | | |  | | | |  |  | | | | |
| Answering questions from the patient's treatment team or his substitute decision-maker regarding disease and treatment | | | | | | | | | | | | | | | | |
| 1. The medical staff jointly responsive to your questions about the disease and treatment? | | | | | | | | | | | | | | | | |
| Ever(1) | | little(2) | | | | | Not least so much(3) | | | | | much(4) | | | Very much(5) | |
| 2. The medical staff and treatment of diseases responsive to questions accompanied you on that treatment? | | | | | | | | | | | | | | | | |
| Ever(1) | | little(2) | | | | | Not least so much(3) | | | | much(4) | Very much(5) | | |  | |
| Section6 | | | | | | |  | | | | | | | | | |
| The patient's right to choose and decide, authority and independence (attention and patient participation in decision making for diagnosis and treatment) | | | | | | | | | | | | | | | | |
| Ever(1) | | little(2) | | | | | Not least so much(3) | | | | much(4) | Very much(5) | | |  | |
| 1. Come to do your own healing processes, you think you are? | | | | | | | | | | | | | | | | |
| Ever(1) | | little(2) | | | | | Not least so much(3) | | | | much(4) | Very much(5) | | |  | |
| 2. Come so far that you suggestions on how to do things related to their treatment, the medical staff did not provide them to suggest you pay attention? | | | | | | | | | | | | | | | | |
| Ever(1) | | little(2) | | | | | Not least so much(3) | | | | much(4) | Very much(5) | | |  | |
| 3. You have a choice in the hospital about your doctor? | | | | | | | | | | | | | | | | |
| Ever(1) | | little(2) | | | | | Not least so much(3) | | | | much(4) | Very much(5) | | |  | |
| 4. Choose your nurse at the hospital about you, you have the right to decide? | | | | | | | | | | | | | | | | |
| Ever(1) | | little(2) | | | | | Not least so much(3) | | | | much(4) | Very much(5) | | |  | |
| 5. Had come to replace the nurse in charge of your care is calling you? | | | | | | | | | | | | | | | | |
| Ever(1) | | little(2) | | | | | Not least so much(3) | | | | much(4) | Very much(5) | | |  | |
| 6. If your answer is yes, do not you been paying attention to this? | | | | | | | | | | | | | | | | |
| Ever(1) | | little(2) | | | | | Not least so much(3) | | | | much(4) | Very much(5) | | |  | |
| 7. The management team has asked your opinion about diagnostic and therapeutic measures? | | | | | | | | | | | | | | | | |
| Ever(1) | | little(2) | | | | | Not least so much(3) | | | | much(4) | Very much(5) | | |  | |
| 8. Ago come to ask about treatment or care how your comment? | | | | | | | | | | | | | | | | |
| Ever(1) | | little(2) | | | | | Not least so much(3) | | | | much(4) | Very much(5) | | |  | |
| 9. If your answer is yes Does the treatment have listened to your comments? | | | | | | | | | | | | | | | | |
| Ever(1) | | little(2) | | | | | Not least so much(3) | | | | much(4) | Very much(5) | | |  | |
| 10. To ask the doctor happened to you other than your doctor about the disease and seek treatment? | | | | | | | | | | | | | | | | |
| Ever(1) | | little(2) | | | | | Not least so much(3) | | | | much(4) | Very much(5) | | |  | |
| 11. If the answer is yes, whether the treatment given to these demands have you? | | | | | | | | | | | | | | | | |
| Ever(1) | | little(2) | | | | | Not least so much(3) | | | | much(4) | Very much(5) | | |  | |
| 12 medical teams are notified that you have the right to a doctor other than your doctor diagnose and treat your patients ask about? | | | | | | | | | | | | | | | | |
| Ever(1) | little(2) | | | | | | | Not least so much(3) | | much(4) | | | | Very much(5) | | |
| **Section6** |  | | | | | | | | | | | | | | | |
| 1. Come from doing certain types of tests or treatment closely and try to refuse medical staff agrees with your opinion? | | | | | | | | | | | | | | | | |
| Ever(1) | | little(2) | | | | | Not least so much(3) | | | | much(4) | Very much(5) | | | |  |
| 2. come forward and agree that you have to ask to change the doctor? | | | | | | | | | | | | | | | | |
| Ever(1) | | little(2) | | | | | Not least so much(3) | | | | much(4) | Very much(5) | | |  | |
| 3. If you do have a certain type of treatment refuse Is it possible complications and consequences you have said? | | | | | | | | | | | | | | | | |
| Ever(1) | | little(2) | | | | | Not least so much(3) | | | | much(4) | Very much(5) | | |  | |
| 4. Before the end of treatment and who do you want to have another center? | | | | | | | | | | | | | | | | |
| Ever(1) | | little(2) | | | | | Not least so much(3) | | | | much(4) | Very much(5) | | |  | |
| 5. If the answer is yes, if you asked the medical team to have success? | | | | | | | | | | | | | | | | |
| Ever(1) | | little(2) | | | | | Not least so much(3) | | | | much(4) | Very much(5) | | |  | |
| 6. If you agree with the request made by the end of treatment, any complications you will notice you have brought consequences of this action? | | | | | | | | | | | | | | | | |
| Ever(1) | | little(2) | | | | | Not least so much(3) | | | | much(4) | Very much(5) | | |  | |
| 7. The personnel department notifies you that you have the right to personal satisfaction for clearance and end of treatment? | | | | | | | | | | | | | | |  | |
| Ever(1) | | little(2) | | | | | Not least so much(3) | | | | much(4) | Very much(5) | | |  | |
| **Section 7:** Patient access to medical records and information of its content | | | | | | | | | | | | | | | | |
| Ever(1) | | little(2) | | | | | Not least so much(3) | | | | much(4) | Very much(5) | | |  | |
| 1. Length of stay in the hospital easily and hassle-free access to their medical records? | | | | | | | | | | | | | | | | |
| Ever(1) | | little(2) | | | | | Not least so much(3) | | | | much(4) | Very much(5) | | |  | |
| 2. Thus far in advance do you want a case study of your medical records, but the staff refused to provide it? | | | | | | | | | | | | | | | | |
| Ever(1) | | little(2) | | | | | Not least so much(3) | | | | much(4) | Very much(5) | | |  | |
|  | | | | | | | | | | | | | | | | |
| Ever(1) | | little(2) | | | | | Not least so much(3) | | | | much(4) | Very much(5) | | |  | |
|  | | | | | | | | | | | | | | | | |
| Ever(1) | | little(2) | | | | | Not least so much(3) | | | | much(4) | Very much(5) | | |  | |
| **Section 8:** Maintaining patient privacy 1. The research and training activities in which they will present you have received satisfaction? | | | | | | | | | | | | | | | | |
| Ever(1) | | little(2) | | | | | Not least so much(3) | | | | much(4) | Very much(5) | | |  | |
| 1. For treatment and care processes that need to touch the body of the patient and back, are you allowed? | | | | | | | | | | | | | | | | |
| Ever(1) | | little(2) | | | | | Not least so much(3) | | | | much(4) | Very much(5) | | |  | |
| 2. Nurses are allowed to take your blood pressure? | | | | | | | | | | | | | | | | |
| Ever(1) | | little(2) | | | | | Not least so much(3) | | | | much(4) | Very much(5) | | |  | |
| 3. In your opinion for treatment and care processes that need to touch the body of the patient and back, his privacy be respected? | | | | | | | | | | | | | | | | |
| Ever(1) | | little(2) | | | | | Not least so much(3) | | | | much(4) | Very much(5) | | |  | |
| 4. Nurses to respect a patient's privacy during injection? | | | | | | | | | | | | | | | | |
| Ever(1) | | little(2) | | | | | Not least so much(3) | | | | much(4) | Very much(5) | | |  | |
| 5. Nurses to respect a patient's privacy when dressing change? | | | | | | | | | | | | | | | | |
| Ever(1) | | little(2) | | | | | Not least so much(3) | | | | much(4) | Very much(5) | | |  | |
| 6. The medical team would be allowed to enter the room from you? | | | | | | | | | | | | | | | | |
| Ever(1) | | little(2) | | | | | Not least so much(3) | | | | much(4) | Very much(5) | | |  | |
| 7. In this hospital to conduct research and educational activities related to your illness you are allowed? | | | | | | | | | | | | | | | | |
| Ever(1) | | little(2) | | | | | Not least so much(3) | | | | much(4) | Very much(5) | | |  | |
| 8. Ever happened that you attended that you are not directly involved in the treatment process, disagree? | | | | | | | | | | | | | | | | |
| Ever(1) | | little(2) | | | | | Not least so much(3) | | | | much(4) | Very much(5) | | |  | |
| If the answer is yes 9. The question was whether to respect your wishes? | | | | | | | | | | | | | | | | |
| Ever(1) | | little(2) | | | | | Not least so much(3) | | | | much(4) | Very much(5) | | |  | |
| 10. Visiting hour’s patient privacy is respected? | | | | | | | | | | | | | | | | |
| Ever(1) | | little(2) | | | | | Not least so much(3) | | | | much(4) | Very much(5) | | |  | |
| **Section 9:** Confidentiality and privacy 1. In your opinion, in the center of the information about your condition, confidential and available only to the treatment team placed | | | | | | | | | | | | | | | | |
| Ever(1) | | little(2) | | | | | Not least so much(3) | | | | much(4) | Very much(5) | | |  | |
| **Section 10:** Patient Education 1. The medical staff training on how to follow the diet prescribed depending on the type of patients you have? | | | | | | | | | | | | | | | | |
| Ever(1) | | little(2) | | | | | Not least so much(3) | | | | much(4) | Very much(5) | | |  | |
| **Section 10: 1.** Of the costs and the right to protest | | | | | | | | | | | | | | | | |
| Ever(1) | | little(2) | | | | | Not least so much(3) | | | | much(4) | Very much(5) | | |  | |
|  | | | | | | | | | | | | | | | | |
|  | |  | | | | |  | | | |  |  | | |  | |
|  | | | | | | | | | | | | | | | | |
|  | |  | | | | |  | | | |  |  | | |  | |
